# Resistance training as a key strategy for high-quality weight loss in men and women

**DOI:** 10.3389/fendo.2025.1725500

**Published:** 2026-01-15

**Authors:** Yair Lahav, Roi Yavetz, Yftach Gepner

**Affiliations:** Department of Epidemiology and Preventive Medicine. School of Public Health, Faculty of Medicine and Health Science, and Sylvan Adams Sports Institute, Tel Aviv University, Tel Aviv, Israel

**Keywords:** aerobic training, body composition, fat free mass, resistance training (RT), weight loss (B)

## Abstract

**Background:**

Preserving fat-free mass (FFM) during weight loss is critical for preventing sarcopenia and maintaining metabolic health. This study examined the effects of resistance training (RT), aerobic exercise (AR), and no exercise (NO) on body composition during a calorie-restricted diet.

**Methods:**

This retrospective cohort study included 304 adults (183 men, 121 women; aged 20–74 years; BMI: 18.5–45 kg/m^2^) who followed a hypocaloric diet and self-selected RT, AR, or NO. The diet was designed to provide an individualized energy deficit of approximately 500 kcal/day, calculated relative to each participant’s measured resting metabolic rate (RMR) and total estimated daily energy expenditure. Body composition was assessed using dual-energy X-ray absorptiometry (DXA), and abdominal circumference (ABC) was measured as a marker of central obesity.

**Results:**

Mean follow-up was 5.1 months ± 0.42 months. In men, total weight loss was similar across groups (NO: − 8.5 kg ± 3.2 kg; AR: − 9.0 kg ± 4.2 kg; RT: − 7.7 kg ± 4.2 kg). However, RT produced the greatest reduction in fat mass (RT: − 8.9 kg ± 4.1 kg; AR: − 7.8 kg ± 3.2 kg; NO: − 5.8 kg ± 2.5 kg) and was the only modality associated with an increase in fat-free mass (RT: + 0.8 kg ± 5.0 kg vs. AR: − 1.1 kg ± 2.0 kg and NO: − 2.8 kg ± 1.4 kg). ABC declined in all groups, with larger reductions in RT (− 9.0 cm ± 3.7 cm) and AR (− 8.0 cm ± 3.2 cm) compared with NO (− 6.1 cm ± 2.4 cm). Among women, weight loss was also comparable between groups (NO: − 7.13 kg ± 3.27 kg; AR: − 6.43 kg ± 3.53 kg; RT: − 5.42 kg ± 3.76 kg). RT produced the greatest fat-mass reduction (RT: − 6.36 kg ± 3.82 kg; NO: − 5.47 kg ± 2.64 kg; AR: − 4.10 kg ± 3.17 kg) and was the only modality that increased fat-free mass (RT: + 0.90 kg ± 1.24 kg). Both NO (− 2.94 kg ± 1.40 kg) and AR (− 0.37 kg ± 1.45 kg) experienced FFM loss. The fat mass (FM)-to-weight loss ratio was lowest in the NO group (0.7 ± 0.2), higher in AR (0.86 ± 0.2), and highest in RT (1.1 ± 0.7; *p* = 0.0002 vs. NO, *p* = 0.0051 vs. AR). ABC reduction correlated strongly with FM loss (*r* = 0.84; *p* = 0.0001), highlighting its utility as a marker of high-quality weight loss.

**Conclusion:**

RT enhances weight-loss quality by maximizing FM reduction while preserving or increasing FFM. Incorporating RT into weight-loss programs may improve long-term weight maintenance and mitigate FFM loss.

## Introduction

The global prevalence of overweight and obesity (body mass index [BMI] ≥ 25 kg/m^2^) is projected to increase from over 2.6 billion individuals in 2020 to more than 4 billion by 2035 (1). Alarmingly, no country has reported a decline in obesity rates, and none are on track to meet the World Health Organization’s goal of maintaining 2010 levels by 2025 (1). Dietary energy restriction remains a cornerstone intervention for weight management, recommended for achieving at least 5% total body weight loss, managing type 2 diabetes (2), reducing adipose tissue, lowering systemic inflammation, and mitigating adverse cardiovascular outcomes (3). However, preserving fat-free mass (FFM) during weight loss is crucial for maintaining physical performance and skeletal muscle integrity. Strategies to minimize FFM loss and promote its retention during caloric restriction are essential for both athletes and the general population.

Central obesity, often assessed via abdominal circumference (ABC), is a stronger predictor of adverse metabolic outcomes than BMI, as it reflects visceral adipose tissue (VAT) accumulation rather than total body mass (4, 5). Epidemiological evidence consistently links increased ABC with heightened morbidity and mortality, underscoring its role as a key indicator of cardiometabolic risk (6, 7). Proper nutrition and exercise are essential for optimizing weight loss composition, promoting preferential fat loss while preserving FFM.

Skeletal muscle mass (SMM), a primary component of FFM, plays a vital role in glucose metabolism (8), independent locomotion via contractile force, and is the main contributor to resting energy expenditure (9). The age-related decline in muscle mass and strength, known as sarcopenia, further exacerbates these risks, emphasizing the importance of maintaining SMM during weight management (10). Current obesity treatments increasingly prioritize “quality” weight loss—defined as reducing fat mass (FM) while preserving lean mass—rather than simply focusing on total weight loss. Research suggests that approximately 25% of weight loss during caloric restriction originates from lean mass, while 75% comes from fat (11). Our previous work demonstrated that even within the normal-weight range, higher adiposity is associated with increased waist circumference and cardiometabolic risk, reinforcing the need to emphasize fat loss over total weight loss, which can be achieved through dietary restriction and exercise (5).

In this study, we examine the effects of resistance training (RT), aerobic exercise (AR), and no exercise (NO) on body composition during caloric restriction, with a focus on FFM preservation and fat loss. We also assess sex-specific differences and evaluate the FM-to-weight loss ratio and ABC changes as markers of weight loss quality. These findings aim to clarify the role of exercise in optimizing body composition during weight loss.

## Materials and methods

### Study population

This retrospective cohort study included 304 men and women who participated in a structured weight loss program at a nutrition clinic in central Israel between 2020 and 2023. Inclusion criteria required participants to be ≥ 20 years old, have a BMI between 18.5 and 45 kg/m^2^, and complete a minimum follow-up period of 1 month. Exclusion criteria included orthopedic limitations that prevented participation in RT or cardiovascular exercise, as well as pregnancy and veganism, because these dietary patterns differ substantially in protein quantity and quality, which could influence body-composition changes and confound the assessment of FFM preservation across exercise modalities. The study was approved by the Ethics Committee of Tel Aviv University (0000607-3).

### Study design and participant assignment

This retrospective cohort study examined the effects of different exercise modalities on body composition during caloric restriction. Participants were recruited from a structured weight loss program at a nutrition clinic in central Israel and were fully informed about the dietary and exercise components of the program. Follow-up meetings were conducted weekly at the start of the program and then reduced to twice per month. The duration of participation ranged from 1 to 18 months, depending on individual progress and adherence. Data collection ended when participants reached a body fat percentage of 14%–24% (men) or 21%–31% (women), based on recommended thresholds for healthy body composition.

At baseline, all participants received education on the benefits of exercise during weight loss, with an emphasis on the “quarter rule”, which states that approximately one-fourth of weight loss is expected to be FFM, with the remaining three-fourths being FM. Although the authors note that this widely cited concept is, at best, an approximation with a limited mechanistic basis regarding the proportion of FFM loss (11), participants received standardized explanations highlighting the role of the prescribed exercise protocol in facilitating reductions in FM while maintaining or improving FFM. Participants self-selected one of three exercise regimens: (1) NO, (2) AR for 150–250 min/week, or (3) RT two to three times per week. All participants followed a hypocaloric diet designed to create a 500-kcal/day energy deficit. Protein intake was prescribed at 1.5 g/kg of total body weight per day. The dietary plan was individually tailored by a registered dietitian based on participants’ food preferences and resting metabolic rate measured at baseline. Participants attended scheduled individual consultations to monitor progress, track weight loss, and adjust their meal plans as needed. Those who selected AR were advised to follow their program at home or at a nearby gym, while those who chose RT were encouraged to train at a gym of their choice following the prescribed program. Exercise adherence was monitored through self-reported logs and follow-up discussions during consultations.

### Dietary guidelines

Subjects received an individualized dietary plan, with energy intake tailored to create a daily deficit of ~ 500 kcal. Energy requirements were determined based on resting metabolic rate (RMR), nonexercise activity thermogenesis (NEAT), and the estimated energy expenditure from each participant’s chosen exercise regimen. The energy restriction was designed to be moderate to minimize lean mass loss, as deficits exceeding 500 kcal/day have been shown to impair muscle protein synthesis (MPS) (12, 13).

Each participant attended a 20- to 30-min personal consultation with a registered dietitian, who remained involved throughout the study to provide guidance and monitor adherence. The dietary plan was adjusted based on individual preferences, progress, and metabolic needs. During each meeting, participants were encouraged to follow their meal plans while maintaining an appropriate macronutrient balance. Individuals who reported difficulty adhering to dietary recommendations were offered weekly meetings for additional support. For specific concerns, participants were contacted via mobile phone or WhatsApp to ensure continuous guidance and adherence.

Participants were advised to consume approximately 1.5 g/kg of total body weight/day of protein, avoid skipping meals, and limit the intake of processed foods. Macro- and micronutrient composition was calculated using specialized dietary analysis software incorporating USDA data.

### Weight loss goal

The primary weight loss goal for participants was to reduce body fat to 24% for men and 31% for women, as these thresholds are associated with prognostic outcomes in coronary heart disease and normal-weight obesity (14–16). Participants who wished to continue reducing body fat beyond these levels, aiming for what is considered a “fitness level” (14), were encouraged to attend follow-up meetings and adhere to the prescribed guidelines to ensure sustainable progress.

### Physical activity guidelines

The RT program included seven upper-body and two lower-body exercises performed using weightlifting machines and free weights. Participants training at home were prescribed a program consisting of nine exercises utilizing free weights and a bench. The initial phase of training involved one to two sets of eight to 15 repetitions without reaching failure, with a progressive increase to three sets performed at near failure. Participants in the RT group were advised to increase repetitions by one to two every two to three workouts until reaching 12–15 repetitions per set. Once this threshold was achieved, additional weight was added, and the cycle was restarted with eight to 10 repetitions.

The AR program consisted of activities such as treadmill or outdoor walking, elliptical training, stationary cycling, and stair climbing. Participants were instructed to exercise at approximately 65% of their maximal heart rate and were encouraged to progressively increase their total weekly exercise duration to 150–250 min. Participants who preferred jogging over walking were advised to transition gradually to minimize injury risk and enhance adherence.

Participants who required additional support in following their assigned exercise regimen were encouraged to schedule weekly meetings to receive guidance and adjustments to their program as needed.

### Anthropometric measurement

Weight was measured to the nearest 0.1 kg using a digital scale (SECA model 400; SECA North America) with participants barefoot and dressed in lightweight clothing (shorts and a T-shirt). Height was recorded to the nearest 0.5 cm using a SECA 274 Free-Standing Wireless 360 Stadiometer (SECA, Hamburg, Germany).

ABC was assessed at the level of the umbilicus using a flexible, nonelastic measuring tape, with measurements taken to the nearest 0.5 cm. Participants were instructed to exhale fully while maintaining an upright posture, and an experienced research assistant performed the measurement. Two waist measurements were recorded, and if a discrepancy of 2 cm or more was observed, a third measurement was taken. The final ABC value was determined as the average of the two closest measurements. Body mass index (BMI) was calculated as weight (kg) divided by height squared (m^2^).

### Body composition measurement

Participants were instructed to arrive at the clinic between 7:00 AM and 10:00 AM after fasting for at least 10 h and refraining from exercise in the preceding 24 h (17).

Body composition was measured using a whole-body scan performed with a narrowed fan-beam dual-energy X-ray absorptiometry (DXA) system (Lunar Prodigy; GE Healthcare, Madison, WI, USA) and analyzed with GE Encore 2011 software (version 13.60, GE Healthcare). To minimize technical variability, the scan followed the Nana protocol, which standardizes participant preparation and positioning, including a 3–4 h fast, voiding before the scan, wearing light clothing, removal of metal objects, and maintaining an anatomically neutral supine posture (12). The DXA system was calibrated daily using manufacturer-provided phantoms, following standard quality control procedures.

All scans were performed in standard-thickness mode, with participants centrally aligned within the scanning area. To ensure consistency, feet were placed in custom-made foam blocks, maintaining a standardized 15-cm distance between them, while hands were positioned in a mid-prone position with a 3-cm gap between the palms and the trunk (18). Baseline assessments, including DXA and ABC measurements, were repeated once participants achieved a 7% to 10% reduction in their initial or current body weight.

### Resting metabolic rate measurement

RMR was measured using indirect calorimetry with a metabolic cart (Quark RMR, Cosmed, Rome, Italy). Participants were instructed to arrive at the clinic in the morning after an overnight fast of at least 12 h, during which only water consumption was permitted, and to refrain from exercise for at least 24 h before the measurement. The use of nicotine products was also prohibited for at least 2 h before testing. To ensure a resting physiological state, participants remained seated at rest for 20 min prior to the measurement, following established guidelines (19).

During the assessment, participants lay awake in a supine position in a quiet room with a stable ambient temperature (22 °C–24°C). RMR was measured using a properly adjusted face mask, with the test lasting a total of 21 min. The first 5 min served as an adaptation phase and were excluded from the final analysis. The mean RMR was calculated from the final 16 min of stable data. If significant movement or sleep occurred during the measurement, the affected period was excluded, and the test was extended to ensure at least 16 min of uninterrupted, stable data. Before each test, the metabolic cart was calibrated for both turbine flow and gas composition according to the manufacturer’s instructions to ensure accuracy and reproducibility.

### Statistical analysis

Statistical analyses were performed to compare body composition outcomes among the three study groups. One-way ANOVA was used to evaluate changes at baseline across groups. Due to some significant differences at baseline, an ANCOVA model was conducted to determine between-group changes in body weight, FM, FFM, and ABC, followed by Bonferroni-adjusted *post-hoc* tests. Associations between continuous variables were evaluated using Pearson’s correlation and linear regression analyses. The normality of continuous variables was assessed using the Shapiro–Wilk test, and homogeneity of variances was examined using the Brown–Forsythe test. All analyses met these assumptions prior to conducting the models. If the normality assumption was violated, a nonparametric Kruskal–Wallis test was conducted instead of ANCOVA, followed by Dunn’s *post-hoc* test for multiple comparisons. All statistical analyses were performed using GraphPad Prism (version 10.4.0 for Windows) and IBM SPSS Statistics (IBM, version 31.0.1.0), with a significance threshold set at *p* < 0.05.

## Results

**Table 1** presents the baseline characteristics of the study population, stratified by gender and study group. The study included a total of 304 participants, comprising 183 men (60.2%) and 121 women (39.8%).

**Table 1:** Baseline characteristics of participants across study groups.

	NO (*n* = 41)	AR (*n* = 88)	RT (*n* = 175)	*P*-value
Men (*n*; %)	17 (42%)	44 (50%)	122 (70%)	< 0.001
Age (year)	42.59 ± 10.8	42.3 ± 12.0	39.7 ± 11.7	0.153
Weight (kg)	88.0 ± 15.8	84.2 ± 17.2	81.7 ± 14.0	0.049
BMI (kg/m^2^)	30.0 ± 4.5	28.5 ± 4.3	27.1 ± 3.3	< 0.001
Body fat (%)	38.2 ± 8.8	36.4 ± 7.0	31.7 ± 8.2	< 0.001
Body fat (kg)	32.2 ± 9.6	29.2 ± 8.4	24.7 ± 7.8	< 0.001
FFM (kg)	54.9 ± 12.4	54.2 ± 12.6	56.3 ± 11.2	0.358
ABC (cm)	104.7 ± 11.1	101.5 ± 10.8	97.4 ± 9.2	< 0.001
Follow-up (m)	4.3 ± 2.6	4.6 ± 2.8	5.6 ± 4.9	0.06

Values are presented as means ± SD. Body mass index (BMI) was calculated as weight in kilograms divided by height in meters squared. Body fat percentage was determined by dual-energy X-ray absorptiometry (DXA). One-way ANOVA was used to determine differences between groups (*p*-values).

*FFM*, fat-free mass; *FM*, fat mass; *ABC*, abdominal circumference.

### Body composition changes in men

**Figure 1:**
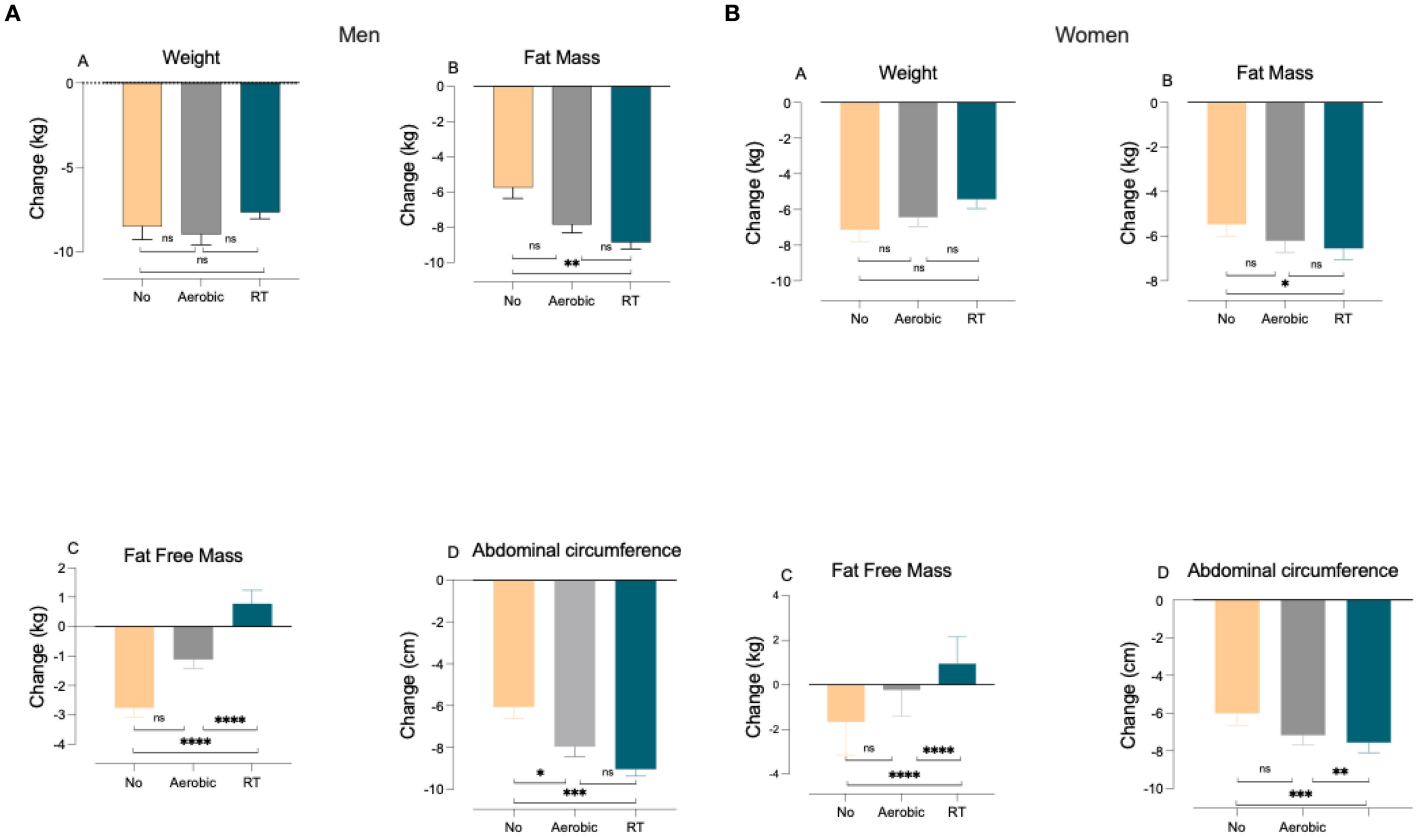
Body-composition changes in **(a)** men and **(b)** women across exercise modalities. Differences between groups were compared using an ANCOVA test with Bonferroni correction. A paired-samples *t*-test was used to compare changes over time. Changes in weight **(A)**, fat mass **(B)**, fat-free mass **(C)**, and abdominal circumference **(D)** among 183 men and 121 women. All data are significant (*p* < 0.05): ^**^p < 0.01; ^***^p < 0.001; ^****^p < 0.0001. Data are presented as mean ± SD.

In men, body weight decreased similarly across the NO (− 8.5 kg ± 3.2 kg), AR (− 9.0 kg ± 4.2 kg), and RT (− 7.7 kg ± 4.2 kg) groups, with no significant between-group differences (**Figure 1A**). All exercise modalities, including no-exercise controls, produced comparable reductions in body weight over the intervention period. FM decreased in all groups, with the greatest reduction observed in the RT group (− 8.9 kg ± 4.1 kg), followed by the AR group (− 7.8 kg ± 3.2 kg) and the NO group (− 5.8 kg ± 2.5 kg). Bonferroni *post-hoc* analysis indicated that RT produced significantly greater FM loss than both the NO and AR groups (both *p* < 0.001), and that AR produced significantly greater FM loss than NO (*p* = 0.038). Overall, RT was the modality associated with the largest fat-mass reduction in men. RT exhibited an increase in FFM (+ 0.8 kg ± 5.0 kg), whereas both the AR (− 1.1 kg ± 2.0 kg) and NO (− 2.8 kg ± 1.4 kg) groups experienced FFM loss. Bonferroni *post-hoc* tests showed that RT differed significantly from both AR and NO (*p* = 0.039 and *p* = 0.006, respectively), while the AR and NO groups did not differ from each other. ABC declined in all groups, with the greatest reductions observed in RT (− 9.0 cm ± 3.7 cm), followed by AR (− 8.0 cm ± 3.2 cm) and NO (− 6.1 cm ± 2.4 cm). Bonferroni *post-hoc* analysis revealed that RT produced significantly greater reductions than both AR and NO (*p* < 0.001 for both comparisons). However, the reduction in the AR group was not significantly different from that in the NO group (**Table 2**, men).

**Table 2A:** Changes in body weight and composition across study groups in men, n=183.

	NO n=17		AR n=44		RT n=122		P value RT vs NO	P value AR vs NO	P value RT vs AR
	Pre	Post	Pre	Post	Pre	Post			
Weight (kg)	97.7±7.9	89.2±6.9	94.8 ± 14.1	85.8 ± 12.3	87.5 ± 10.8	79.8 ± 9.1	ns	ns	ns
FFM (kg)	66.1 ± 6.3	63.3±6.7	64.4 ± 8.6	63.3 ± 8.0	62.6 ± 6.6	63.3 ± 8.6	0.006	ns	0.039
FM (kg)	31.6± 5.4	25.9±5.4	30.4 ± 8.1	22.5 ± 7.2	24.9 ± 8.3	16.1 ± 7.1	< 0.001	0.038	< 0.001
ABC (cm)	108.8 ± 8.0	102.7±7.8	105.7 ± 9.5	97.8 ± 8.8	99.8 ± 9.1	90.8 ± 8.3	< 0.001	ns	< 0.001

**Table 2B:** Changes in body weight and composition across study groups in women, n=121.

	NO n=24		AR n=44		RT n=53		P value RT vs NO	P value AR vs NO	P value RT vs AR
	Pre	Post	Pre	Post	Pre	Post			
Weight (kg)	79.5 ± 15.4	72.4 ± 14.6	72.0 ± 11.7	65.6 ± 10.3	66.0 ± 7.3	60.5 ± 6.8	ns	ns	ns
FFM (kg)	46.9 ± 8.9	45.3 ± 8.9	43.9 ± 5.5	43.7 ± 5.2	41.9 ± 3.9	42.8 ± 4.1	< 0.001	< 0.001	< 0.001
FM (kg)	32.6 ± 11.8	27.1 ± 11.4	28.1 ± 8.7	21.9 ± 8.1	24.1 ± 6.8	17.7 ± 6.1	0.040	ns	ns
ABC (cm)	101.8 ± 12.2	95.8 ± 11.7	97.3 ± 10.5	90.1 ± 10.3	92.0 ± 6.9	84.5 ± 6.5	< 0.001	ns	0.032

Values are presented as mean ± SD. Between-group differences were evaluated using ANCOVA with baseline values as covariates, followed by Bonferroni-adjusted post-hoc comparisons. p-values indicate pairwise differences between groups. FFM, fat-free mass; FM, fat mass; ABC, abdominal circumference.

### Body composition changes in women

**Figure 2:**
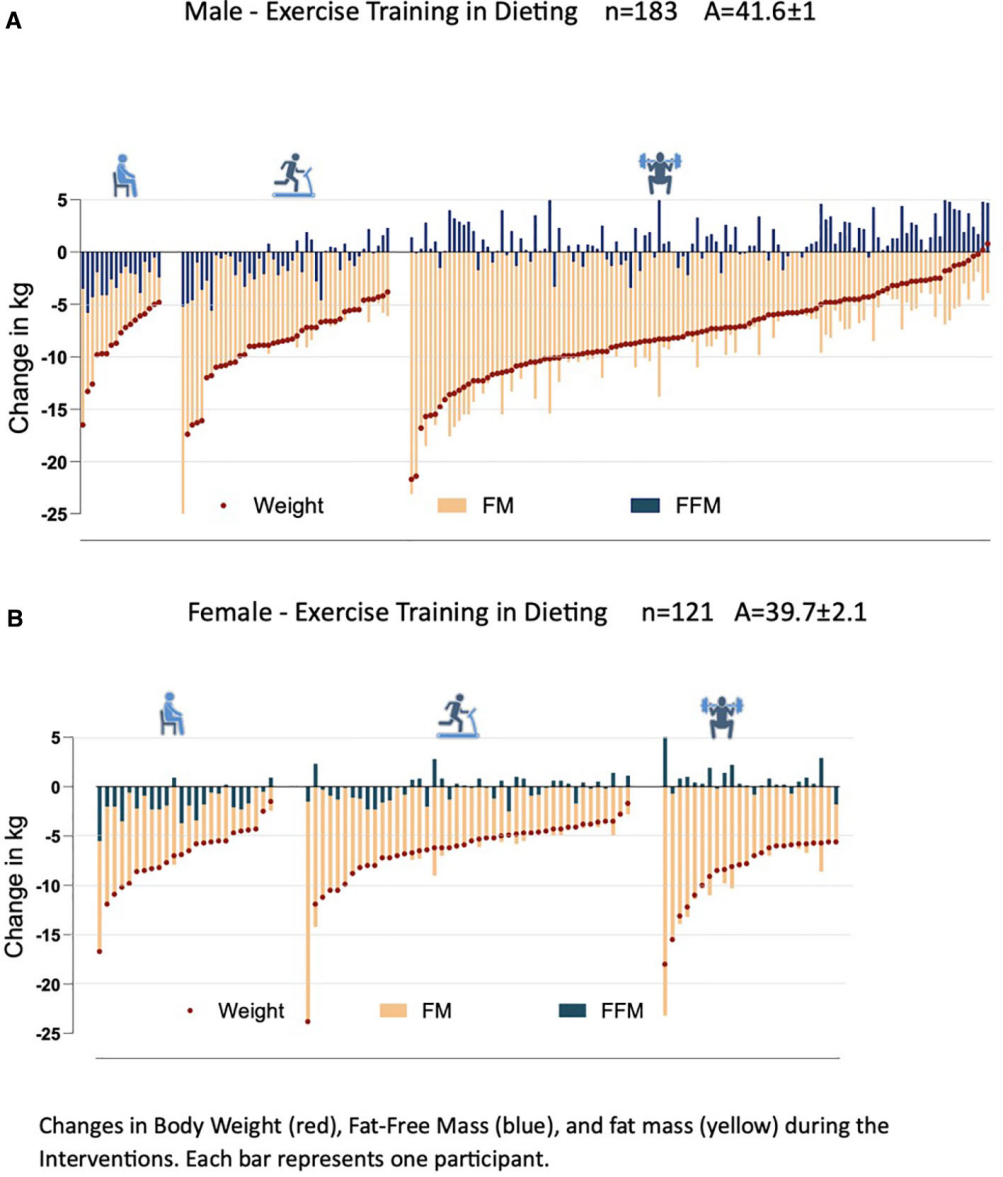
Individual changes in body composition by exercise group: men **(A)** and women **(B)**. Each vertical line represents an individual participant’s change in fat mass (yellow) and fat-free mass (blue). The brown line depicts individual changes in total body weight, sorted from greatest to least weight loss within each group (NO, AR, RT). This figure illustrates the full distribution of individual responses rather than group means.

Among women, weight loss was similar across the NO (− 7.13 kg ± 3.27 kg), AR (− 6.43 kg ± 3.53 kg), and RT (− 5.42 kg ± 3.76 kg) groups, with no evidence that any exercise modality produced greater overall weight reduction during the intervention (**Figure 2B**). FM decreased in all groups, with a significantly greater reduction in the RT group (− 6.36 kg ± 3.82 kg) compared with the NO group (− 5.47 kg ± 2.64 kg; *p* = 0.040). RT was the only modality associated with preservation and an increase in FFM, showing a significant rise of + 0.90 kg ± 1.24 kg. Bonferroni-adjusted pairwise comparisons demonstrated that RT preserved FFM significantly more than both AR (− 0.22 kg ± 1.18 kg) and NO (− 1.66 kg ± 1.49 kg) (*p* < 0.001 for both comparisons). In addition, AR preserved FFM significantly more than NO (*p* < 0.001). Reductions in ABC appeared numerically lower in the RT (− 7.50 cm ± 3.99 cm), AR (− 7.17 cm ± 3.46 cm), and NO (− 6.00 cm ± 3.25 cm) groups. Bonferroni *post-hoc* testing revealed that RT resulted in a significantly greater reduction compared with both NO (*p* < 0.001) and AR (*p* = 0.032). No significant difference was observed between the AR and NO groups (**Table 2**, women).

### Distribution of weight loss components across exercise modalities

The distribution of male and female participants across the different exercise groups illustrates the relationship between weight loss and changes in FM and FFM. Among men, all participants in the NO group experienced FFM loss as part of their weight reduction, and a similar trend was observed in the female NO group. In contrast, in the RT group, only 14.6% of men and 5.7% of women exhibited a substantial loss of FFM (**Figure 2a**, men; **Figure 2b**, women).

### Fat mass contribution to total weight loss

**Figure 3:**
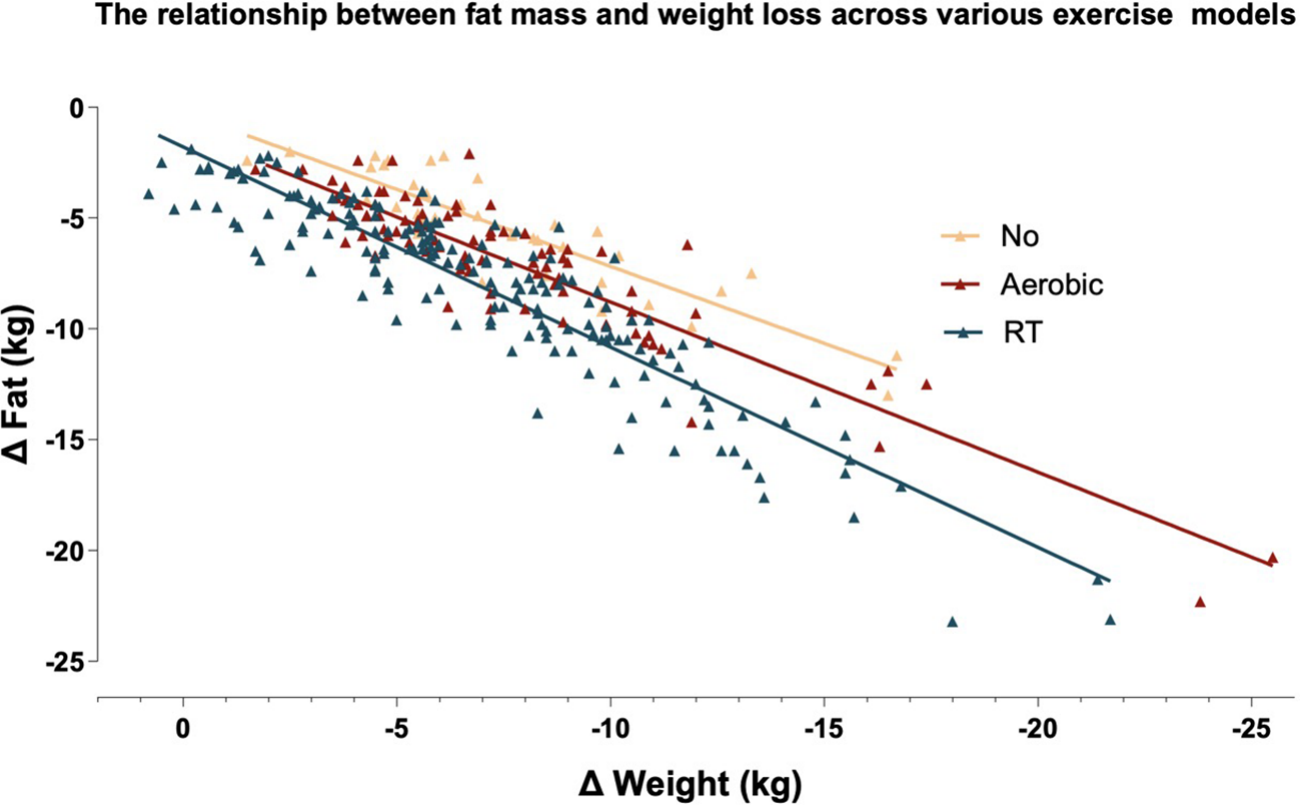
Relationship between weight loss and fat-mass reduction across exercise modalities. This scatterplot illustrates the relationship between body weight loss (*x*-axis) and fat mass reduction (*y*-axis) across three exercise groups: resistance training (RT), aerobic exercise (AR), and nonexercisers (NO). Among nonexercisers, for every 1 kg of weight loss, 0.7 kg ± 0.2 kg is fat. Among aerobic exercisers, for every 1 kg of weight loss, 0.86 kg ± 2.0 kg is fat. Among individuals involved in resistance training, 1.1 kg ± 0.7 kg of weight loss (for both men and women) is attributed to fat loss. This finding is statistically significant (*p* < 0.001).

The proportion of weight loss attributed to FM differed across exercise modalities, as shown in **Figure 3**. The FM-to-total weight loss ratio was highest in the RT group (*R*^2^ = 0.842), followed by the AR group (*R*^2^ = 0.8304) and the NO group (*R*^2^ = 0.7927), suggesting that RT was most effective in promoting preferential FM loss while preserving FFM.

## Discussion

This study demonstrates that RT is the most effective strategy for preserving FFM and improving body composition during weight loss. While total weight loss was similar across all groups, only RT participants gained FFM while reducing FM, indicating body recomposition. AR provided moderate FFM preservation, whereas the NO group experienced significant FFM loss. Additionally, RT led to greater reductions in ABC, reinforcing its role in improving metabolic health and reducing cardiometabolic risk.

### Effect of resistance training on FFM during energy restriction

SMM is crucial for mobility, energy balance, and glucose metabolism, and its loss is a strong predictor of mortality (13). RT is a proven method for maintaining and increasing muscle mass (20)- (21), mitigating the FFM reductions typically observed during caloric restriction (22). Our findings revealed a mean FFM increase of 1.15 kg (95% confidence interval [CI]: 0.82–1.5 kg) in men and 0.94 kg (95% CI: 0.6–1.3 kg) in women in the RT group. Age-related anabolic resistance can limit hypertrophy (23); yet our participants—nonathletes with a mean age of 41 years ± 1.7 years (men) and 39.7 years ± 2.1 years (women)—successfully preserved or gained FFM. Previous studies have shown that 81% of individuals in an energy restriction (ER)-only group and 39% of those in an AR group lost at least 15% of body weight as FFM (22). In contrast, 85% of RT participants in our study gained FFM, and none lost more than 15%, while 50% of AR participants lost FFM.

Several factors likely contributed to this outcome. Progressive overload in RT ensured sustained muscle stimulus, individualized dietary counseling optimized protein intake, and personalized exercise programs enhanced adherence. These findings underscore the importance of RT in weight loss programs, particularly for nonathletic individuals aiming for high-quality weight loss.

According to the well-established rule of weight loss, approximately 25% of weight reduction typically comes from FFM (11). In contrast, our findings demonstrate that RT substantially alters this pattern, preserving a far greater proportion of FFM compared with aerobic training and no-exercise controls. This deviation from the expected composition of weight loss underscores the unique efficacy of RT in protecting lean tissue during caloric deficit. RT significantly preserved and increased FFM in both men and women (*p* ≤ 0.05). While FFM declined in the NO and AR groups, the reduction was smaller in AR, whereas RT participants gained FFM while losing FM. This process, known as body recomposition (18), is often difficult due to suppressed MPS and reduced anabolic signaling during ER (20).

Despite these challenges, FFM increased by 1.15 kg (95% CI: 0.82–1.5 kg) in men and 0.94 kg (95% CI: 0.6–1.3 kg) in women. Age-related declines in anabolic signaling can further impair muscle retention (23). In a previous review of individuals ≥ 50 years with BMI > 25 kg/m^2^, 81% of the ER-only group and 39% of the ER + exercise group lost ≥ 15% of body weight as FFM (23). By contrast, 85% of RT participants gained lean body mass (LBM), and none lost 15%, while 50% of AR participants lost FFM. This may be attributed to the progressive overload in RT, where participants increased weights as much as possible, and to dietary support through consultations, which improved adherence to protein intake.

These findings are particularly relevant as participants were nonathletes with a mean age of 41 years ± 1.7 years in men and 39.7 years ± 2.1 years in women, reinforcing RT as a key strategy for preserving muscle during weight loss.

### Effects of energy restriction without exercise on lean and fat mass

Obese adults losing 15% of body weight typically experience LBM reductions of ~ 3 kg in men and ~ 2 kg in women, often accompanied by SMM loss (19). During ER, downregulation of intracellular signaling proteins involved in MPS can be exacerbated by age, obesity, anabolic resistance, and inflammation (24). In our study, participants in the NO group lost LBM as part of their weight reduction, with LBM comprising 33.9% of total weight loss in men and 23.5% in women. These findings reinforce the well-documented impact of weight loss without exercise, highlighting the importance of structured RT to mitigate muscle loss and maintain metabolic health.

### Abdominal circumference as an indicator of high-quality weight loss

Central obesity is strongly associated with cardiometabolic risk, often more so than BMI. ABC serves as a key marker of visceral adiposity, particularly in individuals with a lower BMI (5). In our study, men in the RT group experienced greater ABC reductions (− 9.0 cm, 95% CI: − 2.0 to − 20.0) compared to NO (− 6.0 cm, 95% CI: − 2.0 to − 10.0). Among women, RT showed a trend toward greater reduction, though not statistically significant.

VAT is the most metabolically harmful fat depot and is closely linked to FM loss across various weight-loss interventions (25). Our findings reinforce that VAT reduction is primarily driven by FM loss rather than the method used to induce the deficit (diet or exercise) (26). **Figure 3** highlights that RT led to greater FM loss relative to total weight loss compared to AR or NO, aligning with prior research linking ABC reductions to VAT, liver fat, and cardiometabolic risk (26).

From a clinical perspective, our results suggest that ER combined with RT is more effective than ER alone or ER + AR in reducing ABC, increasing FFM, and optimizing fat loss. In our cohort, a strong correlation was observed (*r* = 0.84), indicating that each kilogram of FM loss corresponded to an approximately 0.84-cm reduction in ABC. This study-specific relationship highlights the potential utility of ABC as a practical and sensitive marker.

Taking all factors together, body recomposition and high-quality weight loss—characterized by maximizing fat loss relative to total weight reduction—should be the primary target of weight-loss programs to prevent sarcopenia, sarcopenic obesity, and normal-weight obesity, and to promote skeletal health, improve quality of life, and enhance glycemic control and insulin sensitivity across diverse populations (27, 28).

### Strengths and limitations

Our study’s strengths include its large sample size, controlled design, and high adherence rates, which enhance both internal validity and generalizability. The use of DXA, a gold-standard method for body composition analysis, ensured precise assessment of FM and FFM. Additionally, dietary protein intake was standardized to a controlled target, reducing nutritional variability and strengthening the interpretation of body composition outcomes. However, several limitations should be acknowledged. Exercise modality was self-selected rather than randomized, introducing potential self-selection bias and limiting causal inference. Another limitation is that exercise adherence was assessed using self-reported logs, which are susceptible to recall bias and overreporting. This may have introduced some inaccuracy in the estimation of true training adherence. Furthermore, the study did not control for sleep quality, stress, or other lifestyle factors known to influence body composition. Variability in RT and AR training intensity also represents a potential source of heterogeneity. Despite these limitations, the findings robustly support RT as an effective strategy for promoting high-quality weight loss while preserving muscle mass.

## Conclusion

This study underscores the critical role of RT in preserving FFM and optimizing body composition during weight loss. While total weight loss was comparable across groups, only RT resulted in body recomposition, with simultaneous FFM gain and FM reduction. AR provided moderate muscle preservation, whereas the NO group experienced significant FFM loss. Greater reductions in ABC in the RT group further highlight its role in improving metabolic health. These findings reinforce that high-quality weight loss—maximizing FM loss while maintaining muscle mass—should be a key objective. Given the well-established link between muscle loss, metabolic dysfunction, and mortality, integrating RT into weight-loss interventions is essential. Future research should focus on refining RT protocols to further enhance muscle retention and metabolic outcomes across diverse populations.

## Data Availability

The original contributions presented in the study are included in the article/Supplementary Material. Further inquiries can be directed to the corresponding author.
